# Clinical Relevance of Serum Vascular Endothelial Growth Factor and Interleukin-6 in Patients With Colorectal Cancer

**DOI:** 10.4103/1319-3767.80376

**Published:** 2011

**Authors:** Mohammad Alzoghaibi

**Affiliations:** Associate Professor of Physiology, Gastrointestinal Physiology, College of Medicine, King Saud University, Kingdom of Saudi Arabia E-mail: malzoghaibi@ksu.edu.sa; zzoghaibi@gmail.com

Colorectal cancer remains a major health concern in the Western world. It represents the fourth most common cause of cancer-related death.[1] Aging, inflammatory bowel disease, certain hereditary conditions, and a family history of colorectal cancer are the most common risk factors for colorectal cancer. Approximately 6% of colorectal cancers can be attributed to recognizable heritable gene mutations.[2]

Development of human tumors requires a newly formed vascular network.[3] Metastases have shown to be correlated with an increased vascular density; therefore, the inhibition of tumor angiogenesis will arrest tumor growth and decrease metastatic potential. Several growth factors have been shown to stimulate angiogenesis. Vascular endothelial growth factor (VEGF) seems to play a pivotal role in the proliferation and development of endothelial cells, providing blood supply to the tumor cells.[4] Several studies have shown an association between VEGF expression and tumor proliferation both in experimental and human models.[5,6]

Angiogenesis is under the control of several growth factors and cytokines,[7] and the unbalance between stimulator and inhibitor factors is responsible for switching the angiogenic potential of the tumors. VEGF and its receptors are frequently overexpressed in human tumors, including the breast, nonsmall cell lung, colorectal, and prostate cancers,[8] suggesting an association of VEGF with a malignant phenotype. An expression of VEGF mRNA and protein has shown to be associated with tumor progression and poor prognosis of colon carcinoma.[9] The prognosis of advanced colon carcinoma has been attributed to VEGF.[10] Increased levels of VEGF were shown to be related with poor relapse-free and overall survival.[11] Therefore, VEGF may play an important role in determining the risk, prognosis, and survival in colorectal cancer patients.

Patients with chronic inflammation are at increased risk of developing colon cancer.[12] Growing evidence suggests that tumors are promoted by inflammatory signals from the surrounding microenvironment. The immune cells contributing to the pathology of chronic inflammation, such as neutrophils, produce inflammatory cytokines that may influence the carcinogenesis process.[13] Proinflammatory cytokines, such as interleukin-6 (IL-6) and tumor necrosis factor (TNF-α), have been suggested to regulate tumor growth during colitis-associated tumorigenesis.[13] It has been suggested that the signaling pathway via TNF-α/nuclear factor kappaB (NFĸB) may be directly involved in colitis-associated carcinogenesis.[14] Popivanova and colleagues have demonstrated that TNF-α, through its effects on the immune system, plays a critical role in promoting neoplastic transformation.[15] Burstein *et al*. have indicated that cyclooxygenase-2 and nuclear factor kappaB provide an important link between inflammation and cancer and are targets for chemoprevention therapy.[16]

Recent studies have described the importance of IL-6 and its downstream molecules such as STAT3 in colitis-associated colon cancer and their emerging as potential targets for anticancer therapy.[12,17] Chan *et al*.. have demonstrated that plasma levels of TNF receptor 2, but not IL-6, are associated with an increased risk of colorectal cancer.[18] In consistent to the current study, Knüpfer and Preiss have shown that colon cancer patients reveal higher serum IL-6 levels than healthy subjects that were associated with increasing tumor stages and size.[19]

In summary, the current study and others showed that plasma levels of VEGF and inflammatory cytokines, TNF-α and IL-6, may be important diagnostic indicators for colorectal cancer. While some proinflammatory cytokines promote tumor growth, such as IL-6, further studies are needed in order to elucidate the exact role of inflammatory cascade in the cellular level and colitis-associated carcinogenesis.
